# *Ixodes ricinus* and Its Transmitted Pathogens in Urban and Peri-Urban Areas in Europe: New Hazards and Relevance for Public Health

**DOI:** 10.3389/fpubh.2014.00251

**Published:** 2014-12-01

**Authors:** Annapaola Rizzoli, Cornelia Silaghi, Anna Obiegala, Ivo Rudolf, Zdeněk Hubálek, Gábor Földvári, Olivier Plantard, Muriel Vayssier-Taussat, Sarah Bonnet, Eva Špitalská, Mária Kazimírová

**Affiliations:** ^1^Fondazione Edmund Mach, Research and Innovation Centre, San Michele all’Adige, Trento, Italy; ^2^Comparative Tropical Medicine and Parasitology, Ludwig-Maximilians-Universität, Munich, Germany; ^3^Vetsuisse-Faculty, Swiss National Centre for Vector Entomology, Institute for Parasitology, University of Zurich, Zürich, Switzerland; ^4^Institute of Animal Hygiene and Veterinary Public Health, University of Leipzig, Leipzig, Germany; ^5^Institute of Vertebrate Biology, Academy of Sciences of the Czech Republic, v.v.i., Brno, Czech Republic; ^6^Department of Parasitology and Zoology, Faculty of Veterinary Science, Szent István University, Budapest, Hungary; ^7^INRA, UMR1300 BioEpAR, Nantes, France; ^8^LUNAM Université, Oniris, Ecole nationale vétérinaire, agroalimentaire et de l’alimentation Nantes-Atlantique, UMR BioEpAR, Nantes, France; ^9^USC BIPAR, INRA, ANSES – French Agency for Food, Environmental and Occupational Health and Safety, Maisons-Alfort, France; ^10^Institute of Virology, Slovak Academy of Sciences, Bratislava, Slovakia; ^11^Institute of Zoology, Slovak Academy of Sciences, Bratislava, Slovakia

**Keywords:** ticks, *Ixodes ricinus*, tick-borne pathogens, urban habitats, Europe

## Abstract

Tick-borne diseases represent major public and animal health issues worldwide. *Ixodes ricinus*, primarily associated with deciduous and mixed forests, is the principal vector of causative agents of viral, bacterial, and protozoan zoonotic diseases in Europe. Recently, abundant tick populations have been observed in European urban green areas, which are of public health relevance due to the exposure of humans and domesticated animals to potentially infected ticks. In urban habitats, small and medium-sized mammals, birds, companion animals (dogs and cats), and larger mammals (roe deer and wild boar) play a role in maintenance of tick populations and as reservoirs of tick-borne pathogens. Presence of ticks infected with tick-borne encephalitis virus and high prevalence of ticks infected with *Borrelia burgdorferi* s.l., causing Lyme borreliosis, have been reported from urbanized areas in Europe. Emerging pathogens, including bacteria of the order Rickettsiales (*Anaplasma phagocytophilum*, “*Candidatus* Neoehrlichia mikurensis,” *Rickettsia helvetica*, and *R. monacensis*), *Borrelia miyamotoi*, and protozoans (*Babesia divergens, B. venatorum*, and *B. microti*) have also been detected in urban tick populations. Understanding the ecology of ticks and their associations with hosts in a European urbanized environment is crucial to quantify parameters necessary for risk pre-assessment and identification of public health strategies for control and prevention of tick-borne diseases.

## Introduction

Tick-borne infections are arthropod-borne diseases frequently reported worldwide. Ticks are known to transmit a great variety of pathogenic agents producing the highest number of human disease cases compared to other vector-borne diseases in Europe (1, 2). In general, the eco-epidemiology of zoonotic vector-borne diseases is very complex. It depends on the interactions of the vectors with the reservoir hosts and the pathogenic agents, which are modulated by several abiotic and biotic factors that vary in space and time. Certain tick-borne infections have recently been emerging in new regions or re-emerging within endemic sites and create an increasing concern for public health, food security, and biodiversity conservation (3–5). Global warming obviously affects the spread of tick-borne diseases, but climate alone does not determine the geographical distribution of tick species, their population densities and dynamics, the likelihood of their infection with microorganisms pathogenic for humans and animals, nor the frequency of contacts of humans and domestic animals with infected ticks (4, 6, 7). Socio-demographic factors, agricultural and wildlife management, deforestation and reforestation, are known to exert a big impact on the transformation of biotopes, thus affecting tick host assemblages as well as tick infection rates (8–10).

Urbanization as one of the socio-demographic factors has increased worldwide in recent decades (11, 12). Currently, more than half of the world’s population lives in urban areas, and it is expected that 70% will live in urban areas by 2050 (13). Nowadays, more than 75% of Earth’s ice-free lands show evidence of alteration as a result of human residence and land use, with less than a quarter remaining as wildlands. Europe shows the highest level of urbanization worldwide (14). Urbanization, due to restriction of natural areas, is known to dramatically change the composition of wildlife communities and affect the associated tick populations. In European cities, public parks, gardens, peri-urban leisure-time areas, and cemeteries became particularly important places where humans and domesticated animals can encounter potentially infected questing ticks (2).

Urban areas are highly fragmented environments composed of a mosaic of patches of various sizes, vegetation, and land-use types. Urban and peri-urban habitats are generally characterized by lower biodiversity of wildlife species compared to natural ecosystems. Urbanization often produces a certain gradient of homogenization in densely built-up areas, where synanthropic species adapted to urban habitats can be found and where species richness is reduced (15). On the other hand, suburban habitats are also occupied by native species comprising medium-sized mammalian predators and ground-foraging, omnivorous, and frugivorous birds that produce abundant populations there. But urbanization can also result in variation of animal species composition, e.g., by introduction of non-native species that replace native ones (16, 17).

**Figure 1:**
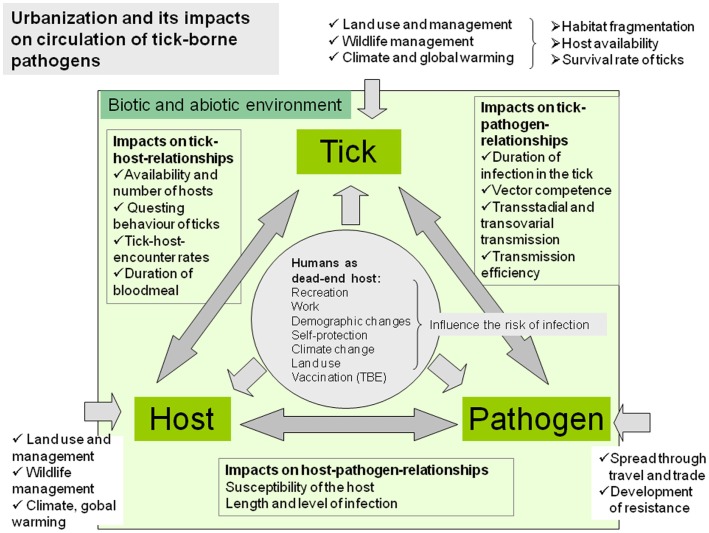
**Complex factors of the biotic and abiotic environment influence the tick–host–pathogen interaction and consequently the occurrence of tick-borne diseases in urban and peri-urban environments**.

Majority of the wildlife species commonly found in urban and peri-urban sites can serve as tick-maintenance hosts and also as reservoirs of tick-borne pathogens (18, 19). Furthermore, the majority of these species are generalists and are able to adapt to the urban and peri-urban environment and reach higher population densities than in natural sites (12, 20, 21). In urban habitats of Europe, rodents (mice, voles, dormice, squirrels, and rats), hedgehogs, shrews, birds, lizards, and companion animals (dogs and cats), but in peri-urban areas also medium-sized and larger mammals like foxes, roe deer, and wild boars, play the major role as tick-maintenance hosts and reservoirs of tick-borne pathogens (19, 22). Adaptation of wild animals to urban environment can also lead to increased contacts with humans and to increased risk of exposure to zoonotic agents. In addition, animal populations in urban areas can show genetic differentiation from wild populations of the same species. Thus urbanization can alter the biology and population densities of ticks and hosts and may lead to increased transmission of pathogens between vectors and urban-adapted hosts (11, 23). Moreover, urbanization is followed by increased mobility of humans, intensive long-distance trade, and new contacts of humans and companion animals with nature, which may contribute to changing of epidemiological and epizootiological conditions in urban and peri-urban areas (12) (Figure 1).

Understanding the ecology of ticks and their association with various hosts in a changing urban and peri-urban European environment is therefore crucial to quantify various parameters necessary for the risk pre-assessment and for the identification of the best public health strategies for tick-borne disease management and prevention. The cascade of events including fluctuations in wildlife community composition and abundance, tick density and emergence, and spread of tick-borne pathogens in various habitat types in Europe are now being modeled as part of the EU FP7 project EDENext^1^. In this review, we focus on *Ixodes ricinus*, one of the principal vectors of pathogens causing arthropod-borne infections in Europe, its associations with hosts and pathogens and risk of infection of humans in urbanized areas.

## *Ixodes ricinus* – Vector of Multiple Pathogens

*Ixodes ricinus* (Acari: Ixodidae) is the most widespread tick species in Europe and transmits several viral, bacterial, and protozoan agents of medical and veterinary importance (8, 24–28).

The distribution area of *I. ricinus* has significantly expanded over the past decades. Recently, the species can be found in more northern areas and habitats at higher altitudes than a few decades ago (29–31). Increase in abundance, habitat expansion, including urbanized areas, and prolongation of the questing activity periods of *I. ricinus*, reported in recent years, are attributed to multiple and interacting factors (19, 26, 32). They include changes in land cover and land use due to alterations in agriculture and forestry management, changes in climate, changes in abundance, and distribution of wildlife due to altering wildlife management, and shifts in socioeconomic factors affecting the rate of exposure of humans to infected ticks (25, 26).

Risk factors associated with *I. ricinus* transmissible diseases can be divided generally into: (i) those directly related to climate change (acting on the tick, the host, or their habitats), (ii) those related to changes in the distribution of tick hosts (which may be a direct or indirect effect of human intervention), and (iii) other ecological changes (also commonly influenced by human intervention) (26).

*Ixodes ricinus* is primarily associated with shrubs and deciduous and mixed forests, with a high abundance of small, medium, and large wild vertebrate hosts. However, as a consequence of changing land use and wildlife management, persistent tick populations and high prevalences of infections with tick-borne pathogens have also been observed in urban and peri-urban sites in many European countries (33–41). *Ixodes ricinus* is a generalist exophilic tick species that is able to feed on over 300 different vertebrate species (42). It has a long-lasting life cycle, involving three active life stages (larvae, nymphs, and adults) that quest and attach to a host and feed on blood for a few days before detachment (parasitic life period) and subsequent molting or laying eggs (females). Each developmental stage requires its specific microhabitat comprising various biotic and abiotic factors. The parasitic on-host life of *I. ricinus* is limited to 3–5 days (larvae), 4–7 days (nymphs), and 7–11 days (females) of feeding on vertebrate hosts, whereas, the non-parasitic off-host life period of all developmental stages can last for several months or years (43). This extremely complex life cycle makes the tick vulnerable to alterations in habitat structure and availability of host animals.

In urban and peri-urban areas, the requirement for high relative humidity (above 80%) for extended periods of time by the off-host stages restricts the occurrence of *I. ricinus* to city parks with litter layers, forest patches, gardens, and cemeteries (22) where the continuous use of water to maintain the vegetation also increases the relative humidity. The other limiting biotic factor for *I. ricinus* in urban environments is the availability of medium-sized and large mammals as hosts of the adults, maintaining persistent and independent tick populations. Shifts in the tick–host associations to, e.g., hedgehogs, foxes, hares, domestic dogs, or cats, due to lack of large mammalian hosts can evoke changes in *I. ricinus*-borne pathogen spectrum, prevalence, and distribution. On the other hand, populations of large animals like deer and wild boar have become more abundant in large city parks and peri-urban areas around European cities, leading to the establishment of tick populations, shift of natural transmission cycles of some pathogens, and increase of the disease risk for humans and domestic animals (19).

## Vertebrate Hosts of Ticks and Tick-Borne Pathogens in Urban Areas

Terrestrial vertebrate hosts are key players in the epidemiology of tick-borne diseases for at least two reasons. Firstly, they serve as maintenance hosts for ticks as a food resource and secondly, as reservoir hosts they are often responsible for the long-term maintenance of pathogens in both natural and urban habitats. Although many reports exist about the presence of pathogens in various hosts or ticks removed from them, the reservoir capacity for each of the pathogens in many cases remains to be experimentally defined. A reservoir host of tick-borne pathogens must fulfill certain criteria: (i) it must feed infected vector ticks, at least occasionally; (ii) it must take up a critical number of infectious agents during an infectious tick bite; (iii) it must allow the pathogen to multiply and to survive in at least certain parts of its body; and last but not least (iv) the pathogen has to find its way into other feeding ticks (44, 45). For this reason, the simple recording of pathogens (or nucleic acid of them) in a vertebrate host is not sufficient for classifying that host as a reservoir, but only a candidate reservoir when physiological and behavioral features may theoretically support pathogen amplification and transmission to the vector, or a simple carrier host, or a dead end host. Similarly, a higher prevalence in ticks removed from the vertebrate host compared to prevalence in questing ticks is only a good indication that the host is a candidate reservoir. However, to unambiguously prove the reservoir status of a host, xenodiagnostic experiments have to be carried out. They involve feeding of specific pathogen-free tick larvae from a laboratory colony on the tested host and the subsequent analysis of them for pathogens after their molt into the next stage. Unfortunately, for most pathogens and hosts, xenodiagnostic experiments have not been performed so far and the key hosts in the natural (and urban) cycle of tick-borne pathogens remain to be tested. The few exceptions are some species of mice, voles, rats, dormice, squirrels, and shrews (see details in Table 1) that had already been proven reservoirs of some tick-borne pathogens.

**Table 1:** **Most important mammal hosts of *I. ricinus* and pathogens transmitted by this tick species with urban or peri-urban occurrence**.

Order	Species	Associated *I. ricinus* stage	Associated pathogens	Reference
Rodentia	*Apodemus flavicollis*	L, N	**TBEV**	(42, 46–50)
			***Borrelia afzelii***	
			***Borrelia burgdorferi* s.s**.	
			***Borrelia spielmanii***	
			***Borrelia miyamotoi***	
			***Cand*. N. mikurensis**	
			*Anaplasma phagocytophilum*	
			*Babesia microti*	
	*Apodemus sylvaticus*	L, N	**TBEV**	(42, 46, 48–52)
			***Borrelia afzelii***	
			***Borrelia burgdorferi* s.s**.	
			***Borrelia spielmanii***	
			***Cand*. N. mikurensis**	
			*Anaplasma phagocytophilum*	
			*Babesia microti*	
	*Apodemus agrarius*	L, N	***Borrelia afzelii***	(42, 50, 53)
			***Cand*. N. mikurensis**	
			*Anaplasma phagocytophilum*	
			***Babesia microti***	
	*Myodes glareolus*	L, N	**TBEV**	(42, 48–50, 54, 55)
			***Borrelia afzelii***	
			***Borrelia burgdorferi* s.s**.	
			***Borrelia miyamotoi***	
			***Cand*. N. mikurensis**	
			*Anaplasma phagocytophilum*	
			*Babesia microti*	
	*Microtus agrestris*	L, N	**TBEV**	(42, 49–51, 56)
			*Borrelia afzelii*	
			*Babesia microti*	
			*Cand*. N. mikurensis	
			*Anaplasma phagocytophilum*	
	*Microtus arvalis*	L, N	*Cand*. N. mikurensis	(53, 55, 56)
			*Anaplasma phagocytophilum*	
			*Babesia microti*	
	*Rattus norvegicus*	L, N	***Borrelia afzelii***	(46, 57)
			***Borrelia spielmanii***	
	*Rattus rattus*	L, N	*Borrelia afzelii*	(46, 50, 57)
			*Anaplasma phagocytophilum*	
	*Eliomys quercinus*	L, N	***Borrelia spielmanii***	(46)
	*Muscardinus avellanarius*	L, N	***Borrelia spielmanii***	(58)
	*Glis glis*	L, N	**TBEV**	(42, 51)
			*Borrelia afzelii*	
	*Sciurus carolinensis*	L, N	***Borrelia afzelii***	(42, 59)
			*Borrelia burgdorferi* s.s.	
	*Sciurus vulgaris*	L, N	**TBEV**	(51, 60, 61)
			*Borrelia burgdorferi* s.s.	
			*Borrelia afzelii*	
			*Borrelia garinii*	
	*Eutamias sibiricus*	L, N	*Borrelia burgdorferi* s.s.	(62)
			*Borrelia afzelii*	
			*Borrelia garinii*	
Lagomorpha	*Lepus europaeus*	L, N, A	*Borrelia burgdorferi* s.l.	(50, 63)
			*Anaplasma phagocytophilum*	
	*Lepus timidus*	L, N, A	*Borrelia burgdorferi* s.l.	(63)
Soricomorpha	*Sorex araneus*	L, N	**TBEV**	(49–51, 63)
			*Borrelia burgdorferi* s.l.	
			*Anaplasma phagocytophilum*	
			*Babesia microti*	
	*Sorex minutus*	L, N	*Borrelia burgdorferi* s.l.	(63)
Erinaceomorpha	*Erinaceus europaeus*	L, N, A	*Borrelia afzelii*	(64–68)
			*Borrelia spielmanii*	
			*Borrelia bavariensis*	
			*Anaplasma phagocytophilum*	
	*Erinaceus roumanicus*	L, N, A	**TBEV**	(47, 64, 69)
			*Borrelia afzelii*	
			*Borrelia bavariensis*	
			*Anaplasma phagocytophilum*	
			*Cand*. N. mikurensis	
Artiodactyla	*Capreolus capreolus*	L, N, A	*Anaplasma phagocytophilum*	(70)
			*Babesia venatorum*	
	*Cervus elaphus*	L, N, A	*Anaplasma phagocytophilum*	(71)
	*Dama dama*	L, N, A	*Anaplasma phagocytophilum*	(71)
Carnivora	*Vulpes vulpes*	L, N, A	*Borrelia burgdorferi* s.l.	(42, 72)
			*Anaplasma phagocytophilum*	
	*Meles meles*	L, N, A	*Borrelia afzelii*	(73)
			*Borrelia valaisiana*	

*Mammal species that are experimentally proven reservoirs for pathogens are in **bold**. *Borrelia burgdorferi* s.l. refers to studies with no species identification (genotyping) of the spirochetes. L, larva; N, nymph; A, adult*.

Urban environments represent many special ecological characters in the complex communities of pathogens, ticks, and hosts. From a public and veterinary health perspective, city parks and peri-urban recreational areas are typical meeting places for humans (their pets) and ticks. Ticks in this respect serve as a bridge for pathogens, connecting reservoir hosts with humans. In addition to the frequent and likely encounter of humans with ticks, vertebrate host communities also differ substantially in many urban habitats compared to natural settings. Some important tick-maintenance and pathogen reservoir hosts (e.g., hedgehogs, squirrels, and songbirds) have no or very few natural enemies within urban environments, thus their populations might reach significantly higher densities compared to natural ones (21, 74). Besides the lack of predators, these urbanized vertebrates can also make use of man-made structures and anthropogenic food resources, like waste and pet food. Hedgehogs are one of the most successful urban adapters reaching up to nine times higher densities in urban areas than in rural areas (74). In Great Britain, red fox density was found at least 10-fold higher in cities than in rural areas (75, 76). The tendency to preserve green spaces inside cities is not only a positive aspect to humans but also for many tick-maintenance and reservoir hosts (12). For these urban dwellers, well established and dense shrubbery in parks offers shelter and nest sites. Furthermore, higher temperatures, especially during winter (heat island effect), are highly beneficial (74). All these factors can lead to an unbalanced vertebrate community that easily provides favorable ecological conditions for tick and pathogen cycles.

### Mammals

Rodents are among the most important maintenance hosts for the subadult stages of *I. ricinus* (77). Furthermore, as pointed out by a recent analysis (78), ecologically widespread, synanthropic species with high density and fast life history such as rodents are often the most competent reservoirs for multi-host pathogens. As a consequence, mice and voles are also known to be important reservoirs for several pathogenic agents like tick-borne encephalitis virus (TBEV), *Borrelia afzelii*, and “*Candidatus* Neoehrlichia mikurensis” (Table 1). In addition to well-established rodent populations in cities, the frequent migration of these animals between human dwellings and natural environments can easily bring infected larvae and nymphs of *I. ricinus* into gardens and houses (79). Fluctuations in rodent densities are very important factors of disease risk (24, 80) with different ecological factors affecting rodent population dynamics in different parts of Europe. However, rodent population dynamics are less studied in urban and peri-urban parks than in natural areas. Rodents can harbor different endophilic (nidicolous) tick species (e.g., *Ixodes trianguliceps* and *I. acuminatus*). These do not pose a direct human hazard since they do not feed on humans. Their co-occurrence with *I. ricinus* on the same rodent, however, can lead to an exchange of pathogens among different tick species.

Little is known about the role of rats (*Rattus rattus* and *R. norvegicus*) in the urban maintenance of ticks and tick-borne pathogens. As one of the most efficient urban adapters, despite the control actions usually undertaken, they might be involved in the urban maintenance of Lyme borreliosis (LB) spirochetes (46, 57, 77). Other urbanized rodents, like garden dormice (*Eliomys quercinus*), hazel dormice (*Muscardinus avellanarius*) (46, 58) and hedgehogs (*Erinaceus europaeus* in Western Europe and *E. roumanicus* in Central and Eastern Europe) are also involved in the urban ecology of LB (Table 1). Hedgehogs have not only a longer life span compared to rodents but they also have the great advantage for ticks being able to feed not only larvae and nymphs but also a considerable number of adults (21, 81). Thus, they can easily maintain stable *I. ricinus* populations in urban areas in the long run (64).

In some cases, anthropogenic introduction of mammals into a new area can lead to the emergence of tick-borne pathogens even previously unknown for that region (12). The gray squirrel (*Sciurus carolinensis*) is native to North America, but an invasive species in the UK that has spread across the country and has largely displaced the native red squirrel (*S. vulgaris*). This species is a frequent urban dweller and can be an indirect source of human tick-borne infections since it has been experimentally shown to be reservoir for *B. afzelii* (59). Siberian chipmunks (*Eutamias sibiricus*) appeared as pets in many European countries but soon these rodents were recorded in urban parks of Rome (82, 83), Geneva (84), Brussels, and in and around many other towns (12). Chipmunks seem to be perfect hosts for subadult *I. ricinus* (85). Pisanu et al. (86) showed that these introduced rodents are more heavily infested by *I. ricinus* than native rodents such as the wood mouse (*Apodemus sylvaticus*) and the bank vole (*Myodes glareolus*). It was also found that the introduced rodent is associated with three species of *B. burgdorferi* sensu lato (s.l.), whereas, the native rodents are associated with only one species (62).

Lagomorphs (hares and rabbits) also inhabit anthropogenic landscapes and serve as blood sources for ticks (79). The European hare (*Lepus europaeus*) and the mountain hare (*L. timidus*) were shown to be not only effective tick-maintenance hosts but also reservoirs for *B. burgdorferi* s.l. (63). The European rabbit (*Oryctolagus cuniculus*) belongs to the most invasive mammalian species and its urban populations can harbor a variety of endo- and ectoparasites including *I. ricinus* (87). These lagomorphs can reach high densities and due to their ability to host adult *I. ricinus* as well, they are able to maintain an infective tick population even in urban and suburban areas where large mammals are not necessarily present. This double epidemiological function (tick-maintenance and reservoir host), which makes them key players in urban cycles of tick-borne pathogens is unique for lagomorphs and hedgehogs.

Bats can also carry different stages of *I. ricinus* ticks, thus they can also transport ticks to urban areas (88). Species especially adaptive in human dwellings, e.g., the lesser horseshoe bat (*Rhinolophus hipposideros*), can serve as tick-maintenance hosts but the role of these flying mammals in the pathogen life cycles remains to be clarified (54). Experimental TBEV viremia was shown in the greater mouse-eared bat (*Myotis myotis*), which is also a common urban inhabitant (51).

Among larger mammalian hosts, which can affect the circulation of tick-borne pathogens in peri-urban areas, roe deer (*Capreolus capreolus*), wild boar (*Sus scrofa*), and red foxes (*Vulpes vulpes*) are particularly important, because they can host all three active life stages of *I. ricinus*, and they increasingly live in urbanized areas (89, 90). Studies on roe deer abundance and movements can provide critical information for predicting tick dispersal and TBEV hazard (91, 92). Deer density is also suggested to be related to the LB incidence (31).

Tick density can be influenced by abundance and distribution of roe deer and red deer (*Cervus elaphus*) (93–95). Roe deer and red deer can inhabit a great variety of tick-infested habitats. Roe deer can even occur in some city parks, e.g., in Munich, Germany (70). Furthermore, the ability of deer to migrate more than 100 km carrying a high number of ticks is also known. This may facilitate the spreading of ticks to other areas (95, 96) and therefore potentially also of tick-borne pathogens from one area to another, although for some pathogen such as *Borrelia* spp., these species dilute the infection rate (97).

Wild boar populations have increased in Europe in recent decades and these animals are well adapted to live in urban and suburban forest areas (98). This species can provide a significant contribution to maintaining tick populations, although its role of reservoir of various tick-borne pathogens is only partially known (98, 99).

Foxes inhabit most urban areas across Europe and population increases have been seen in many European countries, e.g., in Great Britain and Switzerland (100, 101). In a recent study, *I. ricinus* was the most frequently detected tick on foxes in Germany, and all stages of this tick species were found on the animals (90). In Romania, *I. ricinus* infested almost 30% of foxes, indicating that these animals may play a significant role in the epidemiology of tick-borne diseases (102).

Urbanization largely concentrates humans in an area as well as a high number of pets (12). Among these, stray dogs represent an especially effective host for ticks, many of which are *I. ricinus* adults (103). They not only roam in large areas connecting natural and urban habitats, but they also get minimal or no treatment against ticks. Although we have limited knowledge on dogs’ role in the maintenance of tick-borne human pathogens (104–106), as effective hosts for *I. ricinus* adults they certainly contribute to the size of tick populations within gardens, parks, and sub-urban areas. The estimated 100 million free roaming dogs (owned and stray) living in Europe (107) certainly need to be taken into consideration during urban surveillance and control of ticks and tick-borne diseases.

### Birds

Birds play an important role in the introduction of ticks and associated pathogens into urban areas (108, 109). Birds, especially ground-feeding song birds, are important maintenance hosts for larval and nymphal stages of *I. ricinus*. Common urban bird species foraging mostly on the ground and low shrub vegetation, such as common blackbird (*Turdus merula*), song thrush (*Turdus philomelos*), and European robin (*Erythacus rubecula*) were shown to be frequently infested with *I. ricinus* (110–112). More specifically, migratory birds have been shown to carry ticks and pathogens to large distances (113). However, the knowledge on the role of migratory birds in favoring the introduction of ticks and pathogens within new sites is so far very limited (114). Furthermore, earlier onset of spring migration and reproduction with more active ground-feeding activity of birds in the period of questing activities of *I. ricinus* larvae and nymphs may represent an additional risk factor for TBEV spread (115, 116). A recent study highlighted that migratory bird species were infested by more ticks than residents, with urbanized birds being the most parasitized (117). Thus in case of cities being close to bird resting or breeding sites (like cities and towns located on river banks) there is a realistic chance for the introduction and the maintenance of tick-borne pathogens (12). Birds as carriers of infected ticks probably play a role in the geographical spread of pathogens, such as *Rickettsia helvetica, Anaplasma phagocytophilum, Babesia microti*, and *B. venatorum* (118–120).

### Lizards

Lizards have long been known as important hosts for ticks capable of feeding large amounts of immature *I. ricinus* (121) and they can often find suitable habitats in cities. In areas inhabited by lizards they can be as important tick-maintenance hosts as rodents (122, 123). Compared to rodents, however, lizards are more suitable hosts for nymphal *I. ricinus* (as shown by a lower larva/nymph ratio) (124–126). Sand lizards (*Lacerta agilis*), common wall lizards (*Podarcis muralis*), and green lizards (*Lacerta viridis*) are the most common species that can contribute to the urban maintenance of *I. ricinus* populations (122, 123, 125).

The role of lizards in the circulation of tick-borne pathogens has been underestimated compared to that of mammals and birds, but they have been proved to be reservoirs of LB spirochetes (122) and might also be involved in the life cycle of other tick-borne pathogens (124). However, experimental and field studies are needed to shed light on this epidemiological issue.

## Pathogens Transmitted by *Ixodes ricinus*

Among the pathogens transmitted by *I. ricinus*, the western European TBEV subtype (TBEV-Eur), causing tick-borne encephalitis (TBE) (127) and spirochetes of the *B. burgdorferi* s.l. complex, the causative agents of human LB (128) have the greatest impact on human health. *I. ricinus* can also harbor bacteria of the order Rickettsiales that are of rising medical and veterinary importance. Among them, *Anaplasma phagocytophilum* can lead to granulocytic anaplasmosis in both humans and animals (50); the emerging pathogen “*Candidatus* Neoehrlichia mikurensis” can cause severe febrile illness in immunocompromised patients (129) and fever in humans without any primary disease (130); rickettsiae of the spotted fever group (SFG) (*Rickettsia helvetica, R. monacenis*) cause rickettsioses in humans (131). Protozoans of the genus *Babesia*, mainly *B. divergens* and *B. microti*, cause babesiosis in humans, and for *B. venatorum* pathogenicity to humans is suspected (132). The role of *I. ricinus* in transmission of *Bartonella* species (e.g., *B. quintana* and *B. henselae*) causing bartonellosis in humans is suspected (28, 133). *Francisella tularensis*, causing tularemia, and the Q fever agent *Coxiella burnetii* have also been detected in *I. ricinus*, but the role of this tick species in the epidemiology of these diseases is probably not significant (28, 133).

### Tick-borne encephalitis virus

Tick-borne encephalitis is the most important tick-borne arboviral infection of humans in Europe and eastern and central Asia and is caused by the TBEV (Flaviviridae) (134–136). *Ixodes ricinus* is the principal vector for the western European (TBEV-Eur) subtype of the virus (127, 137). TBE is now endemic in 27 European countries (138) and its expansion northward and into higher altitudes has been observed in recent years (137, 139). There is a considerable lack of knowledge in the current fine scale spatial distribution of TBE, including urban areas, thus the risk of infection is still underestimated, especially considering that about two-thirds of human TBE infections are asymptomatic (135).

Incidence of TBE in Europe has been changing in a heterogeneous manner during the last decades, with spatial expansion in some areas and decrease in others (140–142). TBE ecology and epidemiology is expected to be affected considerably by climate change (143) and other drivers like changing in land-use patterns, expansion of forest coverage, increase of abandoned areas, and the creation of new suitable and fragmented landscapes for ticks and hosts within urban areas. Exposure to infected ticks is dependent on several and regionally variable socio-economical factors such as recreational and occupational human activities, public awareness, vaccination coverage, and tourism (26, 94, 144).

The majority of human TBE infections are acquired through bites of infected ticks, more rarely by the alimentary route through consumption of raw milk of infected goats, sheep, or cattle, or unpasteurized dairy products (145–147). As organic markets become more popular, city dwellers also have to be aware of the TBEV infection risk associated with unpasteurized cow and goat milk and milk products.

Tick-borne encephalitis incidence appears to be increasing, including urban areas, partially as a result of improvements in the diagnosis and reporting of TBE cases, but also due to increased exposure of humans to TBE due to outdoor activities. The risk of exposure to TBE was found to be relatively high even in the immediate surroundings of patients’ homes, e.g., in the Czech Republic (148), and an enhanced surveillance of TBE cases in Poland revealed that more than 50% of patients resided in urban areas (149).

Tick-borne encephalitis virus circulates mainly in natural sylvatic cycles involving vector ticks and reservoir hosts. However, due to expansion of urban sites to previously natural habitats and penetration of small and large wild animals into urban areas, reservoir hosts for TBEV as well as large tick-maintenance hosts can be present also in urban and peri-urban sites and thus ensure circulation of the virus there (150). Ticks remain infected throughout their life and it is suggested that they are not only vectors, but also long-term reservoirs of the virus (151). Rodents (*A. flavicollis, A. sylvaticus, M. glareolus*, and *M. arvalis*, see Table 1) are important reservoir hosts for TBEV-Eur (152, 153) and probably may maintain the virus in nature through latent persistent infection (154, 155). Co-feeding tick to tick transmission of TBEV, even in the absence of detectable viremia in these rodent species (156), is crucial to explain the focal distribution of the TBE foci and their potential variation over time (157). Experimental TBEV viremia has been demonstrated also in two lizard species (*L. viridis* and *L. agilis*) often occurring in urban areas (51), but field data on their reservoir competence for TBEV are missing. Migratory birds may play an important role in the geographic dispersal of TBEV-infected ticks, which can contribute to the emergence of new foci of disease, including gardens and urban parks, in case abiotic conditions and the vertebrate host spectrum are favorable for the maintenance of the pathogen (158). Among birds, thrushes (*Turdus* spp.) are the most frequently infested with *I. ricinus* ticks and also carry the most frequently infected ticks (159), however, the prevalence of TBEV-infected bird-feeding ticks is relatively low.

Wild and domestic ungulates, carnivores (foxes and dogs), and hares frequently occurring in peri-urban parks and forest patches within urbanized areas, are important actors in the dynamics of TBE, mainly as tick-maintenance hosts and carriers of infected ticks (160–162). Variation in abundance of roe deer was found to considerably affect TBE risk, depending on the threshold densities of tick, rodent, and large vertebrate populations in the area (31, 91, 92, 163). Ungulates probably do not contribute to the amplification of the virus, but may serve as sentinels to identify TBE foci (163, 164).

Accompanying dogs also represent an important risk factor for humans to acquire TBE. They are accidental hosts, but can become ill with TBE. In addition, during walking in natural forest or hunting activities, dogs come in contact with infected ticks and can carry them home or to urban parks, where they may later infest humans (165).

In general, data on TBEV prevalence in tick populations and seroprevalence in reservoir and sentinel hosts in urban areas and on the circulation of various virus strains in Europe are scarce (166–169). Furthermore, our knowledge on the mechanism favoring TBEV persistence and amplification in urban sites is very limited. TBEV infection rate in ticks is usually very low (<1%) (170–172), but can amount up to 15% in microfoci (173). TBEV-positive *I. ricinus* ticks have recently been detected, e.g., in a highly urbanized region in Southern Poland (estimated pool prevalence ranging from 0.19 to 1.11% for positive locations), suggesting the presence of active foci (174). TBEV-infected *Dermacentor reticulatus* adults were also detected in an urban area (Warsaw) in Poland, with higher prevalence (3.12%) than in natural areas. But our knowledge about the importance of this tick species in TBE epidemiology is still limited (175).

Generally, screening of ticks by PCR cannot be recommended for assessment of human TBE risk and alternative methods of environmental TBEV monitoring should be considered, such as serological long-term monitoring of rodents and other wild and domestic animals, which would serve as sentinel species (169).

### *Borrelia burgdorferi* sensu lato

In little more than 30 years, Lyme borreliosis (LB), which is caused by the spirochete *B. burgdorferi* s.l., has risen from relative obscurity to become a global public health problem and a prototype of an emerging pathogen (176). During this period, we have accumulated enormous progress in knowledge of its phylogenetic diversity, molecular biology, genetics, host interactions, pathogenicity for humans as well as other vertebrate species, and preventive measures including vaccine development. But relatively little is known about public health consequences of LB in terms of eco-epidemiology issues and risk of acquiring infection in suburban and urban habitats.

Lyme borreliosis is the most abundant tick-borne disease of humans worldwide, though it only occurs in the northern hemisphere. LB occurs in North America (from the Mexican border in the south to the southern Canadian provinces in the north), the whole Europe, parts of North Africa (Maghreb), and northern Asia (Russian Siberia and the Far East, Sakhalin, Japan, China, and Korea). The geographical distribution of LB correlates closely with the range of the principal vectors, ticks of the *I. ricinus* complex (177). LB occurs between approximately 35° and 60°N in Europe, and between 30° and 55°N in North America. In countries at the southern limits of the LB range, its incidence decreases rapidly along the north-to-south gradient (178).

The *B. burgdorferi* s.l. complex now comprises up to 19 *Borrelia* species. Of these, only *B. afzelii, B. burgdorferi*, and *B. garinii* are proven agents of localized, disseminated, and chronic manifestations of LB in Europe, whereas, *B. spielmanii* has been detected in early skin disease, and *B. bissettii* and *B. valaisiana* have been detected in samples from single cases of LB (179, 180). The clinical role of *B. lusitaniae* remains to be substantiated.

Principal vectors of *B. burgdorferi* s.l. in Europe, including urban and suburban ecosystems, are two tick species: *I. ricinus* and *I. persulcatus*, the latter only occurring in eastern and north-eastern Europe. Moreover, the occurrence of *I. hexagonus* in the urban environment, due to the presence of suitable hosts, such as hedgehogs, cats, dogs, and foxes in gardens and public parks, could contribute to transmission of LB (65).

The risk of infection is particularly high in deciduous or mixed forest ecosystems or woodlands, along with city parks and urban gardens, especially gardens close to forests (181). The higher risk of contracting LB in the ecotones between forests and arable fields (178) or meadows, although higher densities of infected vector ticks are within forests, is an effect of frequent human presence along the edges of these habitats (182). Also forest fragmentation in suburban areas theoretically poses a greater risk due to enhanced proportion of ecotones (183). Other risks include reforestation (with increased population of forest rodents, but also deer, the principal host of adult vector ticks). For example, in the Czech Republic Zeman and Januska (184) found that LB risk correlated with overall population density of game (red deer, roe deer, mouflon, and wild boar) regardless of rodent abundance. Nevertheless, increased populations of reservoir hosts (forest rodents) usually stimulate the LB incidence.

All activities that increase human contact with ticks present risk for contracting LB, especially recreational (leisure time) activities in forested and urban areas (jogging, berry/mushroom picking, walking, and hiking), seasonal and occasional living by urban residents in country cottages, mowing and clearing of brush around the home in forested areas and gardening. Ownership of pet dogs and cats could also present a relative risk for humans when the pets are frequently parasitized by ticks and the owner tries to remove the ticks (178, 181). Moreover, outdoor employment and work (forestry workers, military personnel in the field, farmers, gardeners, gamekeepers, hunters, and rangers) are at risk. However, in most European countries, occupational exposure generally constitutes only 2% of LB cases (185), whereas, permanent residence in endemic areas with a high prevalence of infectious ticks (e.g., forested peri-urban areas) is a serious risk factor for LB.

Small rodents (*A. sylvaticus, A. flavicollis*, and *M. glareolus*) are regarded as the main reservoir hosts of LB pathogens in urban and suburban habitats across Europe (Table 1). Garden dormice (*E. quercinus*) (186) and hazel dormice (*M. avellanarius*) are especially competent reservoirs of the human pathogenic *B. spielmanii* (46, 58). Important role in the urban maintenance of *B. spielmanii* and *B. afzelii* could also be played by rats (*R. norvegicus* and *R. rattus*) (46, 57, 187). Other key urban players in the maintenance of LB spirochetes are hedgehogs (*E. europaeus* and *E. roumanicus*) (64, 65, 188). Red squirrels (*S. vulgaris*) were found to be heavily infested by ticks and feeding ticks showed high prevalence of infection in enzootic areas in Switzerland (60) and might consequently contribute to maintenance of spirochetes also in urban foci.

Dogs and cats are heavily infested with ticks and might act as hosts (probably not reservoirs) or sentinels for LB. The risk of exposure of dogs to numerous vector-borne pathogens has increased, and close relationship with humans in urban areas poses new concerns for human public health (106).

Ground-foraging bird species such as blackbird (*T. merula*), song thrush (*T. philomelos*), robin (*E. rubecula*), and pheasant (*Phasianus colchicus*) play a unique role in the epidemiology of LB and also contribute to the transmission cycle of *B. burgdorferi* s.l. in urban and suburban areas (189–192). Due to their specific immunity (complement system), certain bird species are resistant to some LB spirochetes but susceptible to others (193). They usually carry *B. valaisiana* and *B. garinii* and transmit these spirochetes to ticks. In 1998, two xenodiagnostic studies clearly defined the reservoir role of birds in the epidemiology of LB, one on a passerine bird, the blackbird (190), the other on a gallinaceous species, and the pheasant (194). However, the reservoir competence of other bird species needs to be clarified. A recent study showed that circulation of LB spirochetes is partly maintained by bird-specific tick species, and bridged by *I. ricinus* to other host types (195).

The role of lizards in the maintenance of *B. burgdorferi* s.l. is still controversial, since several lizard species have been shown to possess a complement with borreliacidal activity (196). However, in some areas LB spirochetes are more prevalent in sand lizards (*L. agilis*) and common wall lizards (*P. muralis*) than in rodents (122). The lizard-associated LB spirochete is *B. lusitaniae*, a genospecies previously thought to occur only in Mediterranean and Central Europe (197), but it was shown that it has a far more widespread geographical distribution involving the green lizard (*L. viridis*), the Balkan wall lizard (*Podarcis taurica*), and the sand lizard (*L. agilis*) (123, 125, 126).

We have reviewed the occurrence of *B. burgdorferi* s.l. in host-seeking urban *I. ricinus* ticks across Europe according to the literature (Table 2). There are also several additional papers demonstrating the presence of borreliae in ixodid ticks collected in (sub)urban areas (198–202). All accessible data show that borreliae in *I. ricinus* ticks collected in urban parks, gardens, or suburban habitats are prevalent approximately at the same rate as in *I. ricinus* ticks living in forests (203). In urban areas, therefore the risk of contacting LB could be as high as in natural environment.

**Table 2:** **Occurrence of *Borrelia burgdorferi* sensu lato in questing *Ixodes ricinus* ticks in urban and suburban areas in Europe**.

Country	City/region (habitat), year	No. of examined ticks	Prevalence^a^	Method	Genomic spp.	Reference
Czech Republic	Prague (U, S)	2,490 N, 143 F, 184 M	2–22%	IFA		(204)
	Prague (U, S) 1994–1997	12,287	3.3–13.3%	IFA		(205)
	Prague 1995–1997	462 N, 173 A	1.9% N, 12.7% A	PCR	Bg 18, Ba 13	(206)
	Brno – outskirts 1988	1,005	3.8% N, 16.4% F, 12.7% M	IFA		(207)
	Brno (U parks) 1992	34 N, 64 F, 65 M	14.7% N, 29.7% F, 30.8% M	DFM		(208)
	Brno-Pisárky (S) 1996–1998	643 N, 123 F, 107 M	10.0% N, 13.8% F, 18.7% M	DFM (and PCR)		(209)
	Brno-Pisárky (S) 2002	243 N, 19 F, 22 M	15.8% N + F + M	DFM (PCR)	Bg 15, Ba 14, Bb 2, Bv 2	(210)
Finland	Helsinki (U, S)	303 N, 189 F, 234 M	32.2% N + F + M	DFM, PCR, BSK	Ba 70%, Bg 25%	(35)
France	Paris (U, S)	360 N, 69 F, 129 M	32% F, 10% N, 20% M	PCR	Ba/Bv 36%, Bg/Bl 60%, Bm 4%*	(211)
Germany	Berlin – West (U, S)	1,414 N, 132 F, 165 M	2.4% N, 9.1% F, 6.1% M (MIR)	BSK		(212)
	Bonn (U, S) 2003	865 N, 241 F, 288 M	17.3% N, 26.6% F, 12.5% M	PCR	Ba 39%, Bg 28%, Bb 16%, Bv 9%	(36)
Hungary	Budapest (parks, forests, and cemeteries) 2013	240 F	40.8%	PCR		(213)
Italy	Imola (U parks) 2006		10.4% N + A	PCR		(214)
Lithuania	Vilnius (city park) 2005	39 A	25%	DFM, PCR	Ba, Bg, Ba + Bg	(215)
The Netherlands	Bijlmerweide (city park) 2000–2002	384 N + F + M	6.8%	PCR	Ba 10, Bb 1, Bv 1	(38)
Poland	Gdansk, Sopot, Gdynia (U, S)	701 N + F + M (164 F, 139 M)	12.4%, 11.6% F, 10.1% M	PCR		(216)
	Szczecin (U, S)	193 N, 22 A	17.7%	DFM		(217)
	Warsaw (U, S), 1996		19.2–31.0%	IFA (PCR)	Bg, Ba, Bv	(218)
	Warsaw (city parks)		6.1%	PCR		(219)
Serbia	Belgrade (U, S) 1996–2005	10,158 N + A	21.9% N + A	DFM (BSK, PCR)	Ba 75%, Bb 22%, Bg 3%	(220)
Slovakia	Bratislava (U, S) 1986–1988	77	7.8%	DFM		(221)
	Košice (U, S) 1991–1995	660 N, 2,904 A	9.2% N, 14.8% A	DFM and IFA		(222)
	Košice, Bardejov (U, S) 2008–2010	670	10.1%	PCR	Ba, Bg, Bv, Bb	(223)
Switzerland	Basel (U, S) 2003	172 N, 35 A	16.4% N + A	PCR		(224)
United Kingdom	London (U parks)	65 F	7.7% F	PCR		(225)

*U, urban; S, suburban; *Ixodes ricinus*: N, nymph; F, female; M, male; A, adult; DFM, dark-field microscopy; IFA, indirect immunofluorescence assay; BSK, cultivation in BSK II medium; Ba, *Borrelia afzelii*; Bb, *B. burgdorferi* s.s.; Bg, *B. garinii*; Bv, *B. valaisiana*; Bl, B. lusitaniae; Bm, B. miyamotoi; MIR, minimum infection rate*.

*^a^Different PCR methods were used that differ in their sensitivity*.

* No sufficient discrimination between Bg and Bl and between Ba and Bv.

We should consider that most studies dealing with eco-epidemiology of LB in patients living in urban areas may have limitation, because not always the exact location (or area) where they acquired the vector tick is known. While popular opinion is that outdoor occupations and hiking are risk activities, several studies have implied that infection is often acquired near the home, during gardening and dog walking associated with increased risk (148, 226–228).

### Anaplasma phagocytophilum

*Anaplasma phagocytophilum* is a small, gram-negative obligate intracellular alpha-Proteobacterium and infects neutrophilic, eosinophilic granulocytes, and monocytes of mammals. There, it replicates within a cytoplasmatic, cell-membrane derived vacuole. *A. phagocytophilum* is transmitted by ticks of the *I. ricinus* complex in the Northern hemisphere and in European countries mainly by *I. ricinus* (50).

The bacterium has been known since the last century to cause diseases in domestic ruminants (229) and since the 1960s in horses (230). The first human case was described in the USA in 1994 (231). The causative agents of the diseases were at the time classified into the granulocytic group of the genus *Ehrlichia*, which contained *E. phagocytophila* as agent of tick-borne fever of ruminants, *E. equi* as agent of equine granulocytic ehrlichiosis and the human granulocytic ehrlichiosis (HGE)-agent. In 2001, a reorganization of the order Rickettsiales, based on homologies in the *16S rRNA* gene, reclassified the granulocytic *Ehrlichia*-group as the new bacterial species *A. phagocytophilum* and the respective diseases were then called granulocytic anaplasmosis (232). Clinical cases are also occurring in dogs and cats, then known as canine and feline granulocytic anaplasmosis (233, 234).

After the first cases appeared in the US in the 1990s, human granulocytic anaplasmosis (HGA) has become one of the most important tick-borne diseases in the US, with an incidence in 2010 of 6.1 cases per 1 million inhabitants^2^. The first human case in Europe was described in the 1990s (235), and around 100 cases have been described since then in several European countries, e.g., in Slovenia, Croatia, Czech Republic, Slovakia, Austria, Latvia, the Netherlands, Norway, Poland, Spain, France, and Sweden (236–252). Seroprevalence rates in humans in Europe are around 1–20% and they fluctuate depending on anamnesis, tick exposure, and age of the patients (253).

Mammalian host species (Table 1) such as wild ruminants (e.g., roe deer, red deer, fallow deer, but also mountain ungulates), small mammals such as rodents and insectivores, but also foxes, bears, wild boars, birds, and reptiles are infected with *A. phagocytophilum* (50). Prevalence rates in wild ruminant species in Europe are generally high, e.g., ranging in roe deer and red deer from around 12% to over 85% (70, 254–256). On the other hand, prevalence rates in small mammals are from 0% to about 20% (50).

*Anaplasma phagocytophilum* is detected with varying prevalences in questing *I. ricinus* ticks, and has been found in Europe in nearly 30 countries. The prevalence ranged, for example, in Norway from 0.4 to 17.1%, in Estonia from 3 to 6.5%, in Slovakia from 1.1 to 8.3%, and in Germany from 1.0 to 17.4% [reviewed in Ref. (50)]. So far, transovarial transmission has not been shown in *Ixodes* ticks. As such, for the current state of knowledge, a reservoir host is necessary to keep up the endemic life cycle of *A. phagocytophilum* in nature.

The discrepancy of a high occurrence of *A. phagocytophilum* in ticks and mammals as well as high seroprevalence rates in Europe in contrast to few clinical cases has been explained by the potential underdiagnosing of the disease, or the potential occurrence of less virulent strains in Europe in comparison to the USA. The discrepancy could also be explained by a higher awareness of US physicians to the disease because in the USA it is a notifiable disease. However, *A. phagocytophilum* shows also genetic heterogeneity and potential differences concerning the potential host tropisms and pathogenicity (118). A potential human pathogenic strain of *A. phagocytophilum* in Europe has been especially suspected to be connected with wild boars. This was confirmed in recent studies (257, 258).

Several studies have investigated the genetic heterogeneity on the basis of several genes such as *16S rRNA, groEL* heat-shock protein, major surface protein coding genes, and the *ankA* gene (255, 259–261). Several distinct clusters were found where, in general, strains derived from domestic animals or ruminants clustered together. Roe deer strains often clustered separately from strains derived from other animals. No evidence was found that wild ruminants are involved in the transmission cycles of potentially pathogenic strains. This was shown again by a recent multi locus sequence typing study (262). However, another study found pathogenic strains associated mostly to ungulates (118).

Furthermore, in a recent large-scale analysis, four *A. phagocytophilum* ecotypes with significantly different host ranges were identified based on *groEL* heat-shock protein gene sequences of various European vertebrate and tick samples (99). So far, all human cases clustered in ecotype I with the broadest host range (including domesticated animals, red deer, wild boar, and urban hedgehogs). Ecotype II was associated with roe deer and some rodents, ecotype III included only rodents. Birds seem to have a different enzootic cycle from all these (ecotype IV). Based on population genetic parameters, ecotype I showed significant expansion, which might have occurred through an increase in either the population of *I. ricinus* ticks, or in the (often urban) vertebrate host species, or in both (99).

Only recently, a HGA case of a German patient has been published having acquired the infection whilst on holidays hiking in Scotland (263). This shows that the risk of contracting this infectious agent can also be in leisure time whilst hiking, or even in the cities whilst being in urban or peri-urban park areas.

In about the last 5 years, considerable research effort has been undertaken in Europe to investigate the epidemiology of *A. phagocytophilum*, especially in urban areas and high prevalences of this pathogen have been found with seasonal and geographic variability. An overview of recent studies investigating questing *I. ricinus* in urban and suburban areas is shown in Table 3. However, when considering *A. phagocytophilum* prevalence rates in ticks, the genetic variability has to be taken into account as not all strains may be pathogenic to humans.

**Table 3:** **Occurrence of *Anaplasma phagocytophilum* in questing *Ixodes ricinus* ticks in urban and suburban areas in Europe^a^**.

Country	City/region (habitat)	No. of ticks posit./examined	Prevalence^b^ (%)	Reference
Austria	Graz (RA)	5/518	1	(264)
Czech Republic	Dvur Kralove (U forest)	8/138	5.8	(265)
	Ostrava (U park)	276 (tested in pools)	9.4	(266)
France	Paris (S forests)	2/558	0.7	(211)
Germany	Hamburg (U RA)	51/1,400	3.6	(267)
	Hannover (U RA)	94/2,100	4.5	(268)
	Bavaria (U parks)	500/5,569	9.0	(269)
	Bavaria (U parks)	103/2,862	2.9	(270)
	Bavaria (U parks)	172/2,800	6.1	(271)
	Leipzig (U, S RA)	47/539	8.7	(55)
	Hannover (U RA)	52/1,646	3.2	(272)
Hungary	Budapest (30 sites: U parks, forests, and cemeteries)	21/240	8.8	(213)
Poland	S forests	18/124; 6/46	14.5; 13.0	(273)
Slovakia	Bratislava (U, S forests)	10/248	4	(265)
	Malacky (U park)	4/101	4	(265)
	Košice (U forest)	10/224	4.5	(265)
	Bardejov Poštárka (S forest)	2/75	2.7	(40)
	Košice Adlerova (S forest)	10/261	3.8	(40)
	Jazero (U forest)	5/91	5.5	(40)
	Košice (S forests)	1,075	1.4–5.5	(274)

*U, urban; S, suburban; RA, recreational area*.

*^a^Negative results not shown*,

*^b^different PCR and real-time PCR methods were used that differ in their sensitivity*.

### *Candidatus* Neoehrlichia mikurensis

“*Candidatus* Neoehrlichia mikurensis” (*Candidatus* N. mikurensis) is a tick-borne pathogen, which is probably transmitted by *I. ricinus* ticks (24). However, transovarial transmission in this tick species has not been reported yet.

Currently, the genera *Wolbachia, Ehrlichia, Neorickettsia, Aegyptianella*, and *Anaplasma* belong to the rickettsial family Anaplasmataceae (232). Most certainly, the new genus “*Neoehrlichia*” will be included in this family in future. The pathogens of this family are intracellular bacteria transmitted by arthropods and may cause severe diseases in humans and animals. For at least three of the five existing genera within this family (*Anaplasma, Ehrlichia*, and *Neorickettsia*) serological cross reactions are not known so far (275). *Candidatus* N. mikurensis is an obligate intracellular gram-negative bacterium, which is characterized by an endothelial cell tropism but it could not be cultivated *in vitro* thus far. Therefore, the status “Candidatus” is still preserved.

A previous study published data on not taxonomically grouped *Ehrlichia* DNA in engorged *I. ricinus* ticks from roe deer in the Netherlands (276). This pathogen was then named after the senior author as “Schotti-Variant” (276). Similar sequencing results were published for *I. ricinus* and *I. persulcatus* ticks from the Baltics in 2001 (277). Between 1998 and 2001, DNA of a pathogen, suggested to be called *Cand*. *Ehrlichia walkerii* spp. nov., was found in engorged *I. ricinus* ticks that fed on asymptomatic patients from Italy (278). In 2003, DNA sequences of this new pathogen were detected in DNA extracted from *I. ricinus* ticks from Germany, followed by first investigations on possible reservoir hosts (279). In 2003, a pathogen was found via examination by conventional PCR in three wild rats (*R. norvegicus*) in China. This examination was followed by DNA sequencing of this pathogen, which was then called the “Rattus Variant” (280). In 2004, DNA of this “new” pathogen was found in 7 out of 15 brown rats from a Japanese isle called Mikura (275). The pathogen was passaged in Wistar rats and first investigations on the ultrastructure and the phylogenetic analysis were done, which lead to the currently valid taxonomic denomination “*Candidatus* Neoehrlichia mikurensis.” The close genetic similarity of the *16S rRNA* and the *groEL* gene puts *Candidatus* N. mikurensis in the family of Anaplasmataceae.

*Candidatus* N. mikurensis was found widespread in *I. ricinus* throughout Europe (281, 282). It could be detected in Italy, France, Sweden, Russia, and other European countries (Table 4). The prevalences ranged between 1 and 11% but focal areas were found with prevalence rates up to 26.6% (49) (Table 4). Furthermore, *Candidatus* N. mikurensis was detected in one out of 126 *I. ricinus* ticks that were collected in Moldavia back in the year 1969 (283) and it was only detected in the genus of *Ixodes* ticks so far (284). Positive ticks were not only found in sylvatic and non-anthropogenic sites but also in urban and peri-urban sites with human influence in Europe (Table 4).

**Table 4:** **Occurrence of *Candidatus* N. mikurensis in questing *Ixodes ricinus* ticks in various habitats in Europe**.

Country	No. of sites, habitat	No. of ticks examined	Prevalence^a^	Reference
Austria	U, S, 2002–2003	518	4.2%	(264)
Czech Republic	U, 2010	69	0.4%	(265)
Denmark	Three sites, S, sylvatic, 2011(+tick DNA from archive)	79^a^	3.8%	(285)
France	Two sites, sylvatic	60	1.7%	(282)
Germany	Ten sites, U, S	542	8.1%	(282)
	U, S, 2008–2009	782	24.2–26.6%	(52)
Hungary	Nine sites, 2007	2,004	n.a. 9 of 35 sites positive	(286)
Italy	U, S, 2006–2008	138	10.5%	(287)
The Netherlands	Three sites, sylvatic	180	8.6%	(288)
	Twenty-one sites, U, S, sylvatic, 2006–2010	5,343	5.6%	(289)
The Netherlands/Belgium	n. a., 2006–2010	2,375	7%	(281)
Russia	S, sylvatic, 1997–1998	295	7.1%	(277)
Slovakia	S, sylvatic, 2006	68	2.9%	(290)
	Ten sites, U, S, sylvatic, 2008, 2010	670	2.4%	(40)
	U, S		1.1–4.5%	(265)
Spain	S, 2013	100	2%	(291)
Sweden	Four sites, sylvatic, 2010–2011	949	4.5–11%	(292)
Switzerland	Eleven sites, U, S, 2009–2010	818	6.4%	(293)
	Four sites, U, S, 2009	1,916	3.5–8%	(294)

*U, urban; S, suburban*.

*^a^Different PCR and real-time PCR methods were used that differ in their sensitivity. n.a., not available*.

Previous studies on potential reservoir hosts revealed that rodents, especially bank voles and yellow-necked mice, but also common voles (*M. arvalis*) were infected at high rates, suggesting a role as reservoir hosts (52, 281, 295, 296), but insectivores were found to be negative for *Candidatus* N. mikurensis thus far (52). Recently, the reservoir role of *Apodemus* mice (*A. flavicollis* and *A. sylvaticus*) and bank voles (*M. glareolus*) has unambiguously been proven in a xenodiagnostic study [(48); Table 1]. Urban hedgehogs (*E. roumanicus*) with high density in a Budapest city park were found to be carriers of *Candidatus* N. mikurensis, indicating that non-rodent reservoirs might be also involved in the maintenance of this pathogen, especially in human dwellings (69). Additionally, *Candidatus* N. mikurensis was detected in dogs from Germany and Nigeria (297, 298).

In the past, the detection of *Candidatus* N. mikurensis in rodents and ixodid ticks was an interesting but only incidental finding without any medical importance (299). In contrast to this assumption, it was recently found in humans (50) with immune deficiency but without being in an occupation group at risk for tick bites over the last decade. *Candidatus* N. mikurensis caused unspecific symptoms such as fever, septicemia, malaise, and weight loss in these patients (300–302). Until October 2012, the first six clinical cases of neoehrlichiosis were the only human cases confirmed by laboratory diagnostic methods. All of these patients suffered from a primary disease, were immunocompromised and came from European countries, such as Germany (301), the Czech Republic (303), Sweden (302), and Switzerland (300). Nevertheless a primary disease is not a necessary precondition to develop neoehrlichiosis as *Candidatus* N. mikurensis could be detected in blood of 7 out of 622 patients from China suffering from fever (130). The authors of these clinical reports emphasize that these seven patients were otherwise healthy and did not suffer from a chronic or immunosuppressive disease. The most recent two human cases were reported in Switzerland, where both patients recovered quickly after a treatment with Doxycycline (294). The data, gained in the last decade, lead to the assumption that *Candidatus* N. mikurensis is an emerging pathogen that might be found by increasing numbers in ticks from sylvatic and urban sites, in small mammals and humans in future (281, 304). Further investigations are needed on the spread, maintenance, and potential reservoir hosts to assess the risk potential of *Candidatus* N. mikurensis.

### Rickettsiae

Rickettsiae are Gram-negative, obligate, aerobic, intracellular bacterial parasites of eukaryotes that survive freely within the cytosol of the host cell, and belong to the family Rickettsiaceae and order Rickettsiales. Rickettsiae are traditionally subdivided into the typhus and the spotted fever group (SFG). SFG rickettsiae are associated with hard ticks (Ixodidae), with the exception of *Rickettsia akari* (mite-borne) and *R. felis* (flea-borne). Hard ticks can transmit them transstadially and transovarially and serve both as vectors and reservoirs of these pathogens. Vertebrates are suspected to serve as reservoirs of rickettsiae, but they may also be accidental hosts and acquire infection by a tick bite (305). However, in a recent xenodiagnostic experiment infected rodents were not able to transmit *R. helvetica* or *R. monacensis* to *I. ricinus* larvae (48).

In Europe, *R. felis, R. typhi, R. prowazekii, R. akari, R. conorii, R. slovaca, R. sibirica mongolotimonae, R. raoultii, R. massiliae, R. aeschlimanni, R. helvetica*, and *R. monacensis* have been implicated in human diseases or reported as emerging pathogens or isolated from vectors or humans (131, 306–308). Furthermore, the candidate species “*Candidatus* Rickettsia kotlanii,” “*Candidatus* Rickettsia barbariae,” or “*Candidatus* Rickettsia vini” have been found in ticks in Europe (309–311). Numerous rickettsiae are regularly associated with ticks and have been called symbionts, microsymbionts, or endosymbionts (living in endocellular symbiosis). However, their potential for pathogenicity is still unknown (312).

The presence of tick-borne rickettsiae has been reported from almost all European countries. The current view on geographic distribution of *Rickettsia* species in the world is summarized by Parola et al. (131).

In Europe, *I. ricinus* ticks are known to carry mainly *R. helvetica* and *R. monacensis*. However, *R. massiliae* was also detected in *I. ricinus* ticks (313). The following rickettsial genotypes were detected only by molecular tools in *I. ricinus* ticks collected in Europe: “*Candidatus* R. vini” was proposed as a new *Rickettsia* spp. detected in *I. arboricola* and *I. ricinus* collected from three different bird species in Spain (311), *Rickettsia* spp. strain Davousti, previously found in *Amblyomma tholloni* ticks in Africa, was detected in *Ixodes* spp. collected from migratory birds in Sweden (314),“*Candidatus* Rickettsia moreli” (GenBank accession numbers Y08784 and Y08785) was detected in *I. ricinus* from Spain, and *Rickettsia* spp. clone KVH-02-3H7 (GenBank accession number GQ849216) was detected in *I. ricinus* in the Netherlands (131).

*Rickettsia helvetica* was first isolated from *I. ricinus* in Switzerland and it was confirmed to be a new member of the SFG rickettsiae in 1993 (315, 316). It has been generally accepted that *I*. *ricinus* is the main vector and natural reservoir of *R. helvetica*. However, *D. reticulatus* ticks were found to be infected with *R. helvetica* in Croatia (317). *R. helvetica* has been detected in questing and bird-feeding *I. ricinus* ticks in at least 24 European countries (131). The prevalence rates vary from 0.5% in a bird conservation island named Greifswalder Oie in the Baltic Sea to 66% in the Netherlands (318, 319). For example, the highest infection rate of *R. helvetica* in *I. ricinus* from Denmark was found in May, followed by July, August, and October (320). The presence of *R. helvetica* was also confirmed in *I. ricinus* in some urban and peri-urban sites in Slovakia, the Czech Republic, Germany, Portugal, Serbia, and Poland (Table 5).

**Table 5:** **Occurrence of *Rickettsia* spp. in questing *Ixodes ricinus* ticks in various habitats in Europe**.

Country	City/region (habitat)	No. examined ticks	Prevalence of *Rickettsia* spp.	Identified species (*n*)	Reference
Czech Republic	Ostrava (U park), 2010	180 N	2.2% (MIR)	14 *Rh*, 6 *Rm*	(266)
		96 A	4.2% (MIR)	
	Proskovice (mixed forest), 2010	1,114 N	3.5% (MIR)	
		83 A	2.5% (MIR)	
France	Paris (S)	360 N, 69 F, 129 M	5.8%	*Rh*	(211)
Germany	Munich, 2006	961 N	1.0%	138 *Rh*, 13 *Rm*	(321)
		1,900 A	7.3%	
	Saarland (RA), 2008–2009	36 N	16.7–47.2%	8 *Rh*	(322)
	Bavaria/Munich (natural alluvial forest), 2008–2009	79 A	21.5%	
	Leipzig/Saxony (coal surface-mining area), 2008–2009	28 N	21.4%	
		100 A	19.0%	
		98 N	8.2–27.6%	
		431 A	9.7%	
	Munich, Regensburg, Ingolstadt, Augsburg, Berg (U parks), 2009–2010	774 L	2.1–9.8%	15 *Rh*, 1 *Rm*	(37)
		1,190 N	6.8%		
		2,495 A	7.5%	77 *Rh*, 4 *Rm*	
		244 L	–	
		742 N	–	180 *Rh*, 8 *Rm*
		1,142 A	–	
	Munich, Regensburg, Lake Starnberg (U, S)	24 L	2.2–7.5%	29 *Rh*,1 *Rm*	(323)
	Lake Starnberg and Lake Ammersee, pastures	500 N	5.0%		
	Augsburg, forest, 2011	889 A	8.7%		
		140 N	15.7%	9 *Rh*
		225A	13.3%	
		139 L	2.2–10.1%	
		120 N	17.5%	9 *Rh*
		79 A	13.9%	
	Hanover (U park), 2010	31 L	16.0%	268 *Rh*	(268)
		1,697 N	25.5%	
		372 A	30.4%	
Poland	Warsaw, national parks and natural areas, 2011	1,147 N 442 A	3.7% (MIR)	38 *Rh, Rm*	(41)
			5.9% (MIR)	
Portugal	Alentejo (safari park), 2006–2009	35 A	82.9%	14 *Rh*, 15 *Rm*	(324)
Serbia	Four natural sites, 2 sites (RA), 2007, 2009	26	23.1%	2 *Rh*, 4 *Rm*	(325)
Slovakia	Bratislava (S forest, cemeteries), 2006–2011	445 N	8.3%	61 *Rh*, 3 *Rm*	(326)
		471 A	10.2%	
	Malacky (U park), 2006–2011	59 N	6.8%	10 *Rh*, 3 *Rm*	
		62 A	14.5%	
	Martin (U park), 2006–2011	3 N	0		
		12 A	16.7%	
	Martinské hole Mts (mountain forest), 2006–2011	276 N	5.4%	6 *Rh*, 2 *Rm*	
		482 A	10.0%	
	Vojka nad Dunajom (RA), 2011–2012	2 N	0	30 *Rh*, 3 *Rm*	(327)
		280 A	11.7%	

*U, urban; S, suburban; RA, recreational area; *Ixodes ricinus*: L, larva; N, nymph; A, adult; MIR, minimum infection rate; *Rh, R. helvetica*; *Rm, R. monacensis**.

In 1999, *R. helvetica* was associated with chronic perimyocarditis in sudden cardiac death in Sweden (328). This species has been cultivated from a patient with subacute meningitis (329). The hypothetical role of *R. helvetica* as an etiological agent of sarcoidosis could not be confirmed (330). The illness is associated with fever, headache, arthralgia, and myalgias and less frequently with rash and/or an eschar (331, 332).

*Rickettsia monacensis* was originally isolated as new species from *I. ricinus* collected in a city park in Germany (333). Phylogenetic analyses of the *16S rRNA, gltA*, and *rompA* gene sequences demonstrated its close relationship with *Candidatus* Rickettsia spp. IRS3 and *Cand. Rickettsia* sp. IRS4 isolated from *I. ricinus* in north-eastern and south-western Slovakia (334, 335). The prevalence rates of *R. monacensis* in *I. ricinus* ticks vary from 0.5% in Germany to 34.6% in Turkey (322, 336). *R. monacensis* has been detected in *I. ricinus* ticks in at least 18 European countries (131). The presence of *R. monacensis* was also confirmed in *I. ricinus* ticks in some urban and peri-urban sites in Slovakia, the Czech Republic, Germany, Portugal, Serbia, and Poland (Table 5). In 2005, *R. monacensis* was identified as a human pathogen in two patients in Spain (in June and September) and latter in one patient in Sardinia, Italy (in April) (337, 338). In addition to fever and flu-like symptoms, the inoculation eschar was identified in an Italian patient, and a generalized rash including the palms and soles was identified in a Spanish patient.

*Rickettsia massiliae* was originally isolated from *Rhipicephalus sanguineus* ticks collected near Marseille, France, in 1992 and then detected in *R. sanguineus, R. turanicus, R. pusillus, R. bursa*, and *I. ricinus* ticks in France, Greece, Portugal, Switzerland, Spain, including islands: Sardinia and Sicily (Italy), the Canary Islands (Spain), Cephalonia (Greece), and Cyprus (131). *R. massiliae* was identified in four *I. ricinus* ticks removed from humans at hospitals in Castilla y León, Spain (313). However, to our knowledge, there are no other studies of this species in urban areas.

### Babesia

*Ixodes ricinus* is the vector of three intraerythrocytic protozoan parasites circulating in Europe and involved in human babesiosis: *B. divergens, B. venatorum* (originally designated *Babesia* spp. EU1), and *B. microti*. To date, no other Piroplasmida affecting humans have been reported to be transmitted by this tick species, even though it feeds on a very large spectrum of hosts, which are potentially infected by several parasite species including numerous other *Babesia* species associated to wildlife or domestic animal diseases. However, the list of potential or known tick-borne pathogens is constantly evolving, either due to: (i) the description of *Babesia* species new for science, (ii) the spread of parasite species previously unknown in Europe, or (iii) the discovery of a *Babesia* species previously restricted to animals but now known to be associated with humans. Thus, emergence or re-emergence of tick-borne diseases leads to the development of unknown health risks (339). Therefore, there is a real concern that tick-borne diseases due to parasites will appear in areas previously free of such diseases, and there is a real necessity of an epidemiological surveillance of the parasitic communities hosted, and potentially transmitted by ticks (340).

Although best known as an animal disease, babesiosis is a zoonotic disease, classified as emerging by some authors. Approximately 50 human cases of babesiosis have been reported in Europe, which is probably underestimated because of a large proportion of asymptomatic infections, as suggested by seroprevalence studies (341). Among the *Babesia* species pathogenic for humans, the bovine parasite *B. divergens* is thought to be responsible for most European cases of human babesiosis (342). However, since 2003, cases of human babesiosis have also been attributed to *B. venatorum* in Austria, Italy, and Germany (343, 344) as well as to *B. microti* in a single case in Germany (341). Whilst the clinical signs of human babesiosis are usually limited to splenectomized patients, two human cases (one attributed to *B. divergens*, the other to an unknown origin) have been detected in immunocompetent patients in eastern France (345). It is also noticeable that, as an example, 0.38% of the French population is splenectomized (346). Moreover, the rising number of HIV-positive individuals and the increasing population of immunocompromised humans, especially in urban areas, may therefore lead to boost the number of human babesiosis cases (341). The proportion of the population at risk of *Babesia* infection is thus higher than previously suspected and *Babesia* spp. likely represents real potential agents of an emerging zoonotic disease and needs increased attention and vigilance.

Besides transstadial transmission, transovarial transmission within ticks is characteristic for most *Babesia* spp. (differentiating them from *Theileria* species), which implies that ticks constitute a real parasite reservoir in the field, facilitating the long-term persistence of *Babesia* species in the ecosystem (sometimes over several tick generations) (347). In Europe, infection rates of *Babesia* spp. in ticks are usually rather low, but published values range from 0.9 to 20% (341).

*Babesia divergens* is a bovine parasite transmitted by *I. ricinus*, and is thought to be responsible for most cases of human babesiosis in Europe (342). This parasite is the most widespread and pathogenic *Babesia* species infecting cattle in northern temperate areas (342). Thus, any urban or peri-urban area where cattle and *I. ricinus* are found is potentially at risk. For example, *B. divergens* has been found in an *I. ricinus* tick collected in an urban park in Germany (37). Recently, the discovery of this parasite in questing *I. ricinus* from a forest area in Eastern France (340), as well as in *I. ricinus* collected from wild cervids in Belgium (348), may suggest that its geographical distribution is increasing, even within forested areas without cattle farms, which would require the existence of reservoir hosts other than cattle. Indeed, it was reported that *B. divergens* is also able to infect ungulates (roe deer, fallow deer, red deer, mouflon, and sheep), splenectomized rats, as well as non-splenectomized reindeer, sheep, and gerbil [see review in Ref. (347)]. Thus, this parasite has been shown to have a wider vertebrate host range than previously thought, leading to a potential risk not only in rural areas but also in peri-urban ones.

*Babesia venatorum*, implicated in human cases of babesiosis in Europe (343, 344), seems to phylogenetically lie in a sister group with *B. divergens* (343), and some serological cross-reactivity between *B. divergens* and *B. venatorum* has been reported (349). Roe deer were strongly suspected to be the wildlife reservoir of this parasite (350, 351) and its transmission by *I. ricinus* was validated both *in vivo* (351, 352) and *in vitro* (353). In addition, *B. venatorum* has been identified in *I. ricinus* in several European countries including Slovenia (354), Switzerland (355), the Netherlands (356), Poland (357), Italy (358), Belgium (359), Germany (37), and France (211, 351), with prevalence varying from 0.4 to 1.3%, demonstrating a wide geographical spread across the continent. Increasing reports of *B. venatorum* in ticks and wild ruminants make this parasite an excellent candidate for the emergence of a new zoonotic tick-borne disease, in particular in the current context of a growing number of wild hosts such as deer. As roe deer is often found even in suburban or peri-urban parks (if they are connected to more natural or semi-natural areas such as forests or rural areas), *I. ricinus* sampled in such places have already been reported as infected by *B. venatorum* (55, 323). This parasite has been detected in 1.3% of questing *I. ricinus* collected in France in a forest located in the South of Paris metropolitan area in the middle of an urban zone (211). Because of its location and the recreational activities available, this forest is visited by over 3 million people every year, emphasizing the public health risk. Similarly, the first detection of *B. venatorum* in Poland has been reported from ticks collected in an urban area (357), and a later study performed in recreational areas, corresponding to peri-urban forest near Warsaw city, showed also the presence of *B. venatorum* in questing *I. ricinus* (360).

Recent molecular phylogenetic investigations have convincingly established *B. microti* as forming a distinct and early diverging clade relative to other *Babesia* species (including the clade containing *B. divergens* and *B. venatorum*) as well as to *Theileria* species (361–363). *B. microti* is responsible for several hundred cases reported yearly in the USA in both spleen-intact and asplenic patient (132). This rodent parasite is known to be transmitted by *I. ricinus*, and now seems to be widely established in Europe, although only one human case has been reported to date (341). The substantial difference in the human pathogenicity of the North-American and European *B. microti* strains need further studies. It has been identified in *I. ricinus* in several European countries such as Switzerland (364), Poland (365), Hungary (366) Slovenia (367), Germany (368), the Netherlands (356, 369), Belgium (359), and France (340). Microtine rodents and probably shrews are the reservoirs of *B. microti* (Table 1). Infectious tick bites are most likely to occur in deciduous woodland and peri-domestic settings (37, 55, 323). Indeed, this parasite was recovered in questing ticks from a forest in Poland that was qualified as “one of the most popular tourist destinations in Poland,” highlighting the risk for humans during recreational activities (360).

### New or neglected tick-borne pathogens: Still unknown bacterial, parasitic, and viral species to be discovered?

Due to advances in molecular biology, new species, strains, or genetic variants of microorganisms are being detected in ticks, resulting in an ever-increasing list of pathogens capable of infecting domesticated animals and humans. Some of them have been linked to human or animal diseases only many years after their first discovery in ticks or animal reservoirs (299). An emblematic example is that of *B. henselae*, the agent of Cat Scratch Disease, known to be transmitted from cat to human by cat scratch (or by fleas). For years, cases of *B. henselae* infection had been described in patients without history of contact with cat without any idea how these people could be infected. By screening pathogens in ticks, *B. henselae* DNA, and RNA were identified in *I. ricinus* (370–373). After many years of debate to know whether *B. henselae* was or was not a tick-borne pathogen, the direct link between tick bites, *B. henselae*, and disease in humans was finally demonstrated (374). Another striking example is the one of *Borrelia miyamotoi*. This *Borrelia* species has been isolated for the first time in Japan in 1995 from *Ixodes* ticks and has been considered as non-pathogenic endogenous tick bacteria until the first human cases of *B. miyamotoi* infection were reported in Russia in 2011 (375). Since then, human infections have been described in the USA and in 2013 in the Netherlands (376–379). In France, *B. miyamotoi* was found to circulate in *I. ricinus* as well as in the bank vole *M. glareolus* (380), and this French genotype was identical to the genotype isolated from a sick person in the Netherlands. These findings have important implications for public health, especially considering that *B. miyamotoi*-positive ticks and rodents were collected from different sites in close proximity to human dwellings. Up to now, no human cases of *B. miyamotoi* infections have been reported in most European countries, however, symptoms caused by *B. miyamotoi* could easily be confused with symptoms caused by other pathogens, which are better known by practitioners, suggesting that surveillance urgently needs to be improved.

A more recent example of neglected pathogens is a new phlebovirus that has been described in humans from northwestern Missouri, USA independently presented to a medical facility with fever, fatigue, diarrhea, thrombocytopenia, and leukopenia, and all had been bitten by ticks 5–7 days before the onset of illness. Electron microscopy revealed viruses consistent with members of the Bunyaviridae family. Next-generation sequencing and phylogenetic analysis identified the viruses as novel members of the phlebovirus genus (381). All these examples demonstrate that new or unexpected tick-borne pathogens are characterized, as soon as they are looked for, in patients bitten by ticks.

## Conclusion

Tick-borne diseases in urban and peri-urban areas represent a rising hazard for public and animal health in Europe. The rapid global changes that planet Earth is facing, especially due to the human ecological footprint, are also affecting the ecology and epidemiology of infectious diseases, including tick-borne diseases. The *I. ricinus* tick being the principal vector of a plethora of viral, bacterial, and protozoan pathogenic microorganisms is showing adaptations to new habitats and ecological conditions. Persistent and potentially increasing populations of this tick species are present in green areas within European cities. Public parks, small forest patches, gardens, and cemeteries are of increasing interest as they represent places where humans, companion, and domestic animals can encounter ticks and be exposed to infected tick bites. The presence of large vertebrates, that serve as tick-maintenance hosts and find conditions to survive and reproduce in the peri-urban environment, reduces the extinction risk of tick populations. Furthermore, majority of tick-maintenance hosts are ecologically classified as generalist species and in many cases serve as reservoirs of a number of emerging zoonotic pathogens, including those transmitted by *I. ricinus*. The combination of urbanization, climate change, and alterations in land-use patterns along with socio-economic factors (outdoor sports and leisure-time activities, gardening, an increased density of pets, and companion animals near human settlements) act in creating favorable conditions for increasing the exposure of humans to ticks, thus favoring the transmission of tick-borne pathogens in urban and peri-urban areas.

Risk communication campaigns aimed at implementing preventive measures against infectious tick bites in urban and peri-urban habitats therefore deserve particular public health efforts. However, several knowledge gaps and lack of quantitative ecological, epidemiological, and socioecological data limit our ability to provide precise quantitative risk pre-assessment. Therefore, more eco-epidemiological research and surveillance specifically focused on the occurrence of ticks, their infection with pathogenic microorganisms as well as on the presence of tick-maintenance and reservoir vertebrate hosts in urbanized areas is urgently needed. Only a multidisciplinary “One-Health” approach integrating research outputs of specialists from different disciplines (veterinarians, zoologists, ecologists, molecular biologists, epidemiologists, physicians, sociologists, and public health experts, etc.), combined with appropriate outreach and dissemination campaigns, can bring success in making urban and peri-urban areas safer from infection by tick-borne pathogens.

## Conflict of Interest Statement

The authors declare that the research was conducted in the absence of any commercial or financial relationships that could be construed as a potential conflict of interest.

## Footnotes

^1^http://www.edenext.eu

^2^http://www.cdc.gov/anaplasmosis
